# Histopathology imaging and clinical data including remission status in pediatric inflammatory bowel disease

**DOI:** 10.1038/s41597-024-03592-7

**Published:** 2024-07-11

**Authors:** Chloe Martin-King, Ali Nael, Louis Ehwerhemuepha, Blake Calvo, Quinn Gates, Jamie Janchoi, Elisa Ornelas, Melissa Perez, Andrea Venderby, John Miklavcic, Peter Chang, Aaron Sassoon, Kenneth Grant

**Affiliations:** 1 Research Institute, Children’s Health Orange County (CHOC), Orange, CA USA; 2 Department of Pathology, CHOC, Orange, CA USA; 3 Department of Pathology, University of California-Irvine (UCI) Medical Center, Orange, CA USA; 4 Schmid College of Science and Technology, Chapman University, Orange, CA USA; 5 Department of Statistics, UCI Donald Bren School of Information and Computer Sciences, Irvine, CA USA; 6 Department of Gastroenterology and Nutrition, CHOC, Orange, CA USA; 7 School of Pharmacy, Chapman University, Irvine, CA USA; 8 Center for Artificial Intelligence in Diagnostic Medicine (CAIDM), UCI, Irvine, CA USA; 9 Department of Radiological Sciences, UCI School of Medicine, Orange, CA USA; 10 Department of Computer Science, UCI Donald Bren School of Information and Computer Sciences, Irvine, CA USA; 11 Department of Pediatrics, UCI School of Medicine, Orange, CA USA

**Keywords:** Pathology, Image processing, Inflammatory bowel disease, Paediatric research, Machine learning

## Abstract

The incidence of inflammatory bowel disease (IBD) is increasing annually. Children with IBD often suffer significant morbidity due to physical and emotional effects of the disease and treatment. Corticosteroids, often a component of therapy, carry undesirable side effects with long term use. Steroid-free remission has become a standard for care-quality improvement. Anticipating therapeutic outcomes is difficult, with treatments often leveraged in a trial-and-error fashion. Artificial intelligence (AI) has demonstrated success in medical imaging classification tasks. Predicting patients who will attain remission will help inform treatment decisions. The provided dataset comprises 951 tissue section scans (167 whole-slides) obtained from 18 pediatric IBD patients. Patient level structured data include IBD diagnosis, 12- and 52-week steroid use and name, and remission status. Each slide is labelled with biopsy site and normal or abnormal classification per the surgical pathology report. Each tissue section scan from an abnormal slide is further classified by an experienced pathologist. Researchers utilizing this dataset may select from the provided outcomes or add labels and annotations from their own institutions.

## Background & Summary

Inflammatory bowel disease (IBD) can be a complex and difficult disease to diagnose, with several contributing elements. Current diagnostic methods involve assessment of clinical, endoscopic, radiologic, and histologic parameters. Histopathological examination of tissue collected during endoscopy can support, challenge, or help refine a suspected IBD diagnosis. Distinguishing features of the tissue, including location, focality, active inflammation, granulomas, and crypt architecture, facilitate making a diagnosis and parsing IBD into its two classic subtypes: ulcerative colitis (UC) and Crohn’s disease (CD).

Once a child is diagnosed with IBD, treatment goals include mucosal healing of the gastrointestinal tract, reaching growth potential, limiting medication toxicities, and optimizing quality of life. Long-term consequences of chronic inflammation can be devastating, especially in children, who may have active disease for a very long time. Therefore, achieving prompt, durable remission is crucial. Corticosteroids are a classic therapy and can induce remission in IBD. However, long-term use of steroids is associated with adverse effects including growth delay, adrenal suppression, infection risk, bone demineralization, poor wound healing, and facial swelling.

Artificial intelligence (AI) and machine learning (ML) applications that use biomedical imaging have demonstrated value in addressing the current limitations of clinical decision-making for care of patients with IBD. A ML computer-aided diagnosis system for predicting disease activity in adult IBD from endoscopy images achieved diagnostic sensitivity of 74%, specificity of 97%, and accuracy of 91%^1^. A region-based convolutional neural network (R-CNN) takes only 18 seconds to provide a disease classification score from a 20-minute colonoscopy^2^. An image-based AI approach based on tissue histology outperformed a comparator group of trained observers in predicting prognosis for colorectal disease^3^. The algorithms used to train a CNN in differentiating normal vs. abnormal or ulcerative mucosa from capsule endoscopy images showed 91% accuracy^4^.

The motivation for the collection of this dataset comes from clinician-directed research aimed at leveraging AI to predict pediatric IBD outcomes from diagnostic imaging and clinical attributes, to optimize treatment for individual patients. Existing studies for improving IBD outcomes have focused on the treatment of adult disease. Furthermore, the limited number of studies that have focused on pediatric IBD assess drug efficacy and/or outcomes using only structured data^5^, often from a single time-point. The application of AI in IBD care is limited by the availability of longitudinal and phenotyping data, which are required for robust, generalizable models^6^. The dataset we are providing directly addresses this gap. Despite the known value of histologic findings from endoscopy for diagnosis, status evaluation, and therapeutic decisions, histologic indexes validated in pediatric cohorts are still lacking^7–9^. Finally, using AI and ML to classify disease phenotype based on mucosal histopathological indices, which are presently underutilized^7^, is likely to improve the chances of sustained disease remission.

The data included in this repository consist of image scans of hematoxylin and eosin (H&E)-stained histology slides and associated structured clinical data. Slide section scans are provided as-is without any initial image pre-processing, normalization, or cropping. To our knowledge, this is the first publicly available pediatric IBD slide image dataset. This dataset does not limit studies to one or two outcome variables at a singular level. Labels are provided at the patient, slide (anatomic site), and section (tissue slice) levels. Furthermore, researchers can use the data for more than diagnostic predictions. Studies utilizing histopathology datasets of expert-selected regions of interest, rather than images of all tissue on a slide, are not realistic for streamlined clinical application. This data is suitable for use in the development of computational tools capable of processing large amounts of imaging data such that analyses can be performed with minimal user intervention. This could result in a clinically feasible, realistic, and attractive application for real-time use.

## Methods

The retrospective study associated with the provided dataset is considered minimal risk and was approved by CHOC’s In-House Internal Review Board (IRB), IRB number 2111186. A waiver of informed consent was granted. CHOC IBD Program patients less than 22 years of age as of January 1, 2021, who underwent endoscopic biopsy during the period from 2014 to 2022, were considered for this study.

### Data acquisition

The data included in this repository consists of image scans of H&E-stained histology slides and associated structured clinical data. Data collection occurred in two phases. First, manual chart review was performed to extract IBD diagnosis (CD or UC), as well as identify encounter dates at three time-points: endoscopy visit, 12-week visit, and 52-week visit, according to the standard of clinical care. Steroid use, steroid name (if applicable), remission status, and nutrition status were recorded at the second and third time-points: 12-week and 52-week visits, respectively. Mental health risk is only provided for patients at the 12-week time-point since these values were missing for all patients at the 52 week time-point. Second, all tissue sections from all slides associated with each patient’s endoscopy visit were scanned.

Histopathology from endoscopic biopsies is a standard part of IBD diagnostic evaluation. Tissue collection occurred at a single time point during the endoscopy encounter at the first time point. Surgical pathology report findings were recorded for each scanned slide and confirmed by the pathologist. A convenience sample was selected based on availability of slide specimens. The first 18 patients with slide scanning completed, and variable values for both 12- and 52-week encounters, are included in this dataset.

The three time-points of structured data collection were: (1) endoscopy encounter at which the tissue was biopsied, (2) IBD outpatient encounter that occurred closest to 12 weeks following endoscopy, and (3) IBD outpatient encounter that occurred closest to 52 weeks following endoscopy. Table 1 provides a summary of data elements that were captured at each visit.

**Table 1:** Data elements acquired for each time point.

First time point	Second time point	Third time point
Endoscopy visit	12-week visit	52-week visit
•Biopsy tissue	•Steroid use status	•Steroid use status
	•Steroid name (if applicable)	•Steroid name (if applicable)
	•Remission status	•Remission status
	•Nutrition status	•Nutrition status
	•Mental health risk	

Steroid use status and name of steroid (budesonide, prednisone, prednisolone, methylprednisolone, hydrocortisone) were collected for the 12- and 52-week time-points. Patients on topical, inhaled, or intravenous (IV) steroids were coded as “none” for steroid use due to lack of clinical relevance. A patient was considered to be steroid-free at the time of the visit if they had ceased taking the steroid medication at least one day prior to the visit, or if they had never previously been on steroid medication.

Remission status was recorded at the 12-week and 52-week time-points. Remission status was determined by the provider per ImproveCareNow (ICN) guidelines for assessment of IBD^10^. The standard for remission was met if within the week preceding the encounter, the patient had minimal or no symptoms secondary to IBD. Specifically,

- No unexplained weight loss, no abdominal masses or tenderness, no toxic appearance.
- Either no fistula or a non-inflamed, indolent fistula with no or minimal drainage.
- If laboratory tests were performed, either normal results or results with minimal transient abnormalities.
- Either no symptoms or mild symptoms (abdominal pain, diarrhea, bloody stools, fatigue, limited daily activity) on ≤ 2 occasions that resolved spontaneously.

Nutrition status was recorded at the 12-week and 52-week time-points. Nutrition status describes the patient’s nutritional status at the time of their visit per ICN guidelines. “At risk” indicates mild to moderate malnutrition, “In failure” indicates severe malnutrition, “Obese” indicates BMI for age ≥95th percentile, “Satisfactory” indicates that the patient did not fall in the other categories at their visit.

Mental health risk is provided for the 12-week time-point and indicates whether there were any psychosocial factors determined by the provider to significantly impact the patient’s medical care at the 12-week visit.

### Slide scanning

**Fig. 1:**
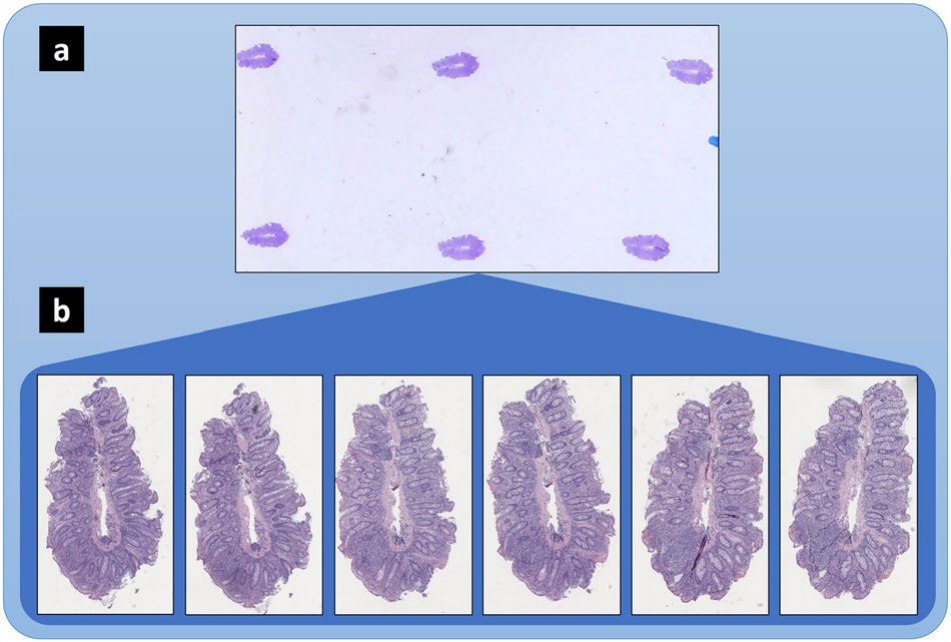
The dataset includes 167 slides and 951 tissue section images from 18 patients. (**a**) Representative whole-slide macro image (≈9 slides per patient). (**b**) Six tissue section images obtained from the whole-slide in (**a**) (≈6 tissue sections per slide).

Slides were scanned using a MikroScan digital pathology scanner at 40x magnification, 0.227–0.258 micrometers per pixel (MPP) resolution. All tissue sections on each slide were scanned individually with the associated computer user interface by creating a bounding rectangle around each individual tissue section. At least three focal points were added to each rectangle prior to scanning to focus the microscope on the tissue and avoid interference from artifacts. Figure 1 provides slide and tissue section scan details at the specimen and dataset levels.

## Data Records

Slide imaging and structured data associated with 18 pediatric IBD patients treated at CHOC from 2014 to 2022 are included in this repository. Each patient was assigned an anonymous identification number. Image deidentification included removing slide labels and macros from each ScanScope Virtual Slide (SVS) image file. Macro images provide a low-resolution snapshot of all sections on the slide. Labels and macros were removed since many were found to contain protected health information (PHI). Patient demographics and IBD diagnoses are listed in Table 2.

**Table 2:** Patient summary statistics.

Variable	n (%) or median (IQR)
Gender	
Female	7 (36.8%)
Male	11 (61.1%)
Ethnicity	
Hispanic or Latino	3 (16.7%)
Not Hispanic or Latino	15 (83.3%)
Diagnosis	
Crohn’s disease	8 (44.4%)
Ulcerative colitis	10 (55.6%)
Age in years at biopsy	13.6 (11.6, 15.1)
12-Week Steroid Use	
None	13 (72.2%)
Steroid	5 (27.8%)
52-Week Steroid Use	
None	12 (66.7%)
Steroid	6 (33.3%)
12-Week Remission	
Yes	13 (72.2%)
No	5 (27.8%)
52-Week Remission	
Yes	15 (83.3%)
No	3 (16.7%)
12-week visit elapsed weeks	9.9 (9.3, 11.9)
52-week visit elapsed weeks	51.3 (48.0, 55.4)

IQR: Interquartile range.

### Experimental design

An associated Excel file for this imaging data set contains three sheets, one for patient-level details, a second for image-level details, and a third listing column names and descriptions.

#### Patient-level details

The patient sheet contains patient identification number, gender, age in years at biopsy, IBD diagnosis, number of weeks between biopsy encounter and 12-week encounter, 12-week remission status, 12-week steroid name (for patients on steroids at that time), 12-week nutrition status, 12-week mental health risk, number of weeks between biopsy encounter and 52-week encounter, 52-week remission status, 52-week steroid name (for patients on steroids at that time), 52-week nutrition status, number of slides, and number of scans. A 52-week mental health risk variable is not included since all patients were missing values for this variable.

#### Image-level details

Biopsy pathology results indicating the treating pathologist’s designation (abnormal vs. normal) at the site level are included in the image-level Excel sheet, which lists all section scan images included in this dataset. A normal classification was assigned to a site if there were no significant histopathologic abnormalities. Generally, any “-itis” was classified as abnormal. Exceptions were mild inactive gastritis and reactive esophagitis, which were classified as normal. These findings are not usually IBD related, and there is a high prevalence of these findings in patients at CHOC, even patients without IBD. Images were classified as abnormal gastritis if they included any active/acute gastritis (even focal) or any chronic gastritis with at least subepithelial band-like lymphoplasmacytic infiltration or cluster(s) of >10 plasma cells within the lamina propria. Further parsing (normal vs. abnormal) of the data at the section level is provided for tissue sections from abnormal slides. Not all tissue sections on an abnormal slide were classified as abnormal.

Abnormal tissue section images were additionally classified as containing active inflammation (mild, moderate, severe), granuloma, and/or chronic changes/architectural distortion. Mild active inflammation was assigned if the section contained neutrophils within the lamina propria and/or cryptitis. Moderate active inflammation was assigned if the section contained crypt abscesses. Severe active inflammation was assigned if the section contained inflamed granulation tissue, erosion, or ulceration. Chronic changes/architectural distortion was assigned if the section contained branched glands, crypt drop-out, lymphoplasmacytosis within the lamina propria, and/or eosinophilia. Granuloma was assigned if the section contained at least one granuloma.

Active inflammation is present in 318 sections, granulomas are present in 18 sections, and chronic changes are present in 272 sections. Active inflammation, granuloma, and chronic changes are not mutually exclusive phenotypes. All tissue sections on a slide come from the same sample. Usually, the 6 sections are taken at 3 different levels about 200 microns apart: 3 sets of 2 consecutive sections. However, although all sections on a slide are from the same tissue sample, not all sections on an abnormal slide are classified as abnormal and not all sections from a single slide are phenotypically identical. For instance, it is possible for some sections on a slide to contain both active inflammation and chronic changes, while the other sections on the same slide only contain chronic changes. Although the tissue appears very similar, using several slices from the same tissue sample is standard pathology practice because differences exist within the sample. Subtle features in the tissue sections make the section level classification tasks interesting and clinically useful. Table 3 lists the number of slides and section scan images classified as normal vs. abnormal, as well as the number of slides and scans associated with remission within normal and abnormal classifications and abnormal phenotypes.

**Table 3:** Slide and section scan classification.

Classification	Number of slides (% of all)	Number of section images (% of all)
Normal	97 (58.1%)	558 (58.7%)
12-Week Remission	73	433
52-Week Remission	75	454
Abnormal	70 (41.9%)	393 (41.3%)
12-Week Remission	53	281
52-Week Remission	61	312
**Abnormal Phenotype**	**Number of slides (% of all)/(% of abnormal)**	**Number of section images (% of all)/(% of abnormal)**
Inflammation	63 (37.7%)/(90%)	318 (33.4%)/(80.9%)
12-Week Remission	50	258
52-Week Remission	55	272
Granuloma	4 (2.4%)/(5.7%)	18 (1.9%)/(4.6%)
12-Week Remission	2	12
52-Week Remission	4	18
Chronic changes	50 (29.9%)/(71.4%)	272 (28.6%)/(69.2%)
12-Week Remission	38	213
52-Week Remission	42	228

### Data access

Tissue scan images and the associated Excel file are available on the Cell Image Library (CIL) at http://cellimagelibrary.org/pages/Project_20483^11^. The dataset is licensed under Creative Commons Attribution License CC BY and can be downloaded using the PHP script provided at https://github.com/CRBS/CIL_RS/wiki/Download_CHOC_dataset. The entire dataset is 241.5 GB. Each scan image will be downloaded to a zip file. Once the images are extracted from the zip files, they can be linked to patient and scan attributes listed in the Excel file per a standardized file-naming convention. All scan images have the*.tif* file extension and are named according to the following:

PatientID_SpecimenLetter_BiopsySiteLetters_SectionLetter.tif. Note that patient IDs are not consecutive; there are 18 patients, but patient ID numbers range from 01 to 23. The specimen letter is included to prevent duplicate names in cases where multiple pieces of tissue were taken from the same site. Table 4 lists the biopsy site name and associated letters utilized to name each tissue-section image file. Possible biopsy sites that did not occur in this dataset were duodenal bulb and mid-esophagus. The section letter is representative of the section’s location within the tissue sample. The 6 sections were taken at 3 different levels about 200 microns apart: 3 sets of 2 consecutive section pairs; A and B, C and D, and E and F. For slides with only 3 sections (A, B, and C), each section was taken about 200 microns apart.

**Table 4:** Biopsy site key.

Biopsy Site Letters	Biopsy Site
DD	Duodenum
ST	Stomach gastric
SA	Stomach antrum
SB	Stomach body
ED	Esophagus distal
EP	Esophagus proximal
TI	Terminal ileum
TD	Terminal ileum distal
TP	Terminal ileum proximal
CE	Cecum, cecum colon
CA	Colon ascending, right colon, hepatic flexure
CT	Colon transverse
CD	Colon descending, left colon, splenic flexure
CS	Colon sigmoid, rectal sigmoid colon
CR	Colon random
RE	Rectum

## Technical Validation

At the slide-level (anatomical site-level), normal and abnormal tissue was determined based on the surgical pathology report, which was evaluated by the pathologist assigned to that patient’s case as part of their care team. At the section-level, initially a single pathologist with 10 years of experience, labelled all tissue sections from abnormal slides as normal vs. abnormal and as containing active inflammation, granuloma, and/or chronic changes. Due to the time intensive burden for labeling the tissue section scans, an additional pathologist with over 30 years of experience labelled a subset of patients for comparison. Labels from both pathologists are provided in the structured data in separate columns. The interobserver reliability score was calculated for the subset, which consisted of scans from 9 patients, using Cohen’s Kappa^12^, *κ*. Kappa was calculated for sections that came from abnormal slides in the subset; a total of 174 sections from 87 abnormal slides were labeled by the second pathologist. Possible *κ* values range from −1 to 1 with values from 0 to 0.2 indicating slight agreement, >0.2 to 0.4 indicating fair agreement, >0.4 to 0.6 indicating moderate agreement, >0.6 to 0.8 indicating substantial agreement, and >0.8 to 1 indicating almost perfect or perfect agreement^13^. Interobserver reliability scores with 95% confidence intervals for each phenotype are provided in Table 5. Weighted *κ* is provided for active inflammation since there is an ordinal relationship between the classes: absent, mild, moderate, and severe. Interobserver rater results are given in Table 6.

**Table 5:** Interobserver reliability for additionally labelled subset.

	*κ* (95% CI)	Weighted *κ* (95% CI)
Abnormal vs. normal	0.799 (0.626, 0.973)	
Active inflammation	0.839 (0.769, 0.909)	0.934 (0.902, 0.965)
Granuloma	0.860 (0.704, 1.000)	
Chronic changes	0.643 (0.519, 0.767)	

κ: Cohen’s Kappa Statistic; CI: confidence interval.

**Table 6:** Interobserver rater results for additionally labelled subset.

		Rater 2			
**Abnormal vs normal**		Abnormal		Normal	
**Rater 1**	Abnormal	158		1	
	Normal	4		11	
**Active inflammation**		Mild	Moderate	Severe	Absent
**Rater 1**	Mild	84	3	0	1
	Moderate	0	31	0	0
	Severe	0	0	24	0
	Absent	14	0	0	17
**Granuloma**		Present		Absent	
**Rater 1**	Present	10		2	
	Absent	1		161	
**Chronic changes**		Present		Absent	
**Rater 1**	Present	105		12	
	Absent	15		42	

A project manual was used to streamline biopsy site classification. If a biopsy site or classification listed in the pathology report was unclear to the person scanning the associated slide, the slide was flagged, then properly labelled once the pathology report was checked by the pathologist. Scans found to be out of focus or containing pervasive artifacts were rescanned following recalibration of the scanner. The more user-intervention that is required in the form of manual selection and preparation, the less likely the models developed from utilizing the data will be robust or clinically useful. Therefore, only tissue scans determined to be unusable due to significant dust or air bubbles beneath the coverslip were excluded from the dataset.

## Usage Notes

The images are in SVS TIFF format with the*.tif* file extension. They can be viewed and annotated using standard, opensource slide-viewing software, such as Aperio ImageScope, OpenSlide^14^, and QuPath^15^; and can be pre-processed for model development using OpenSlide-Python or TiffSlide^16^. TiffSlide should be used for interpreting slide levels as OpenSlide does not detect slide level properties for this dataset.

When using the dataset for deep learning experiments, we suggest breaking each scan into overlapping patches, resizing the patches to dimensions that are feasible for model input, then parsing the patches into useable and unusable sets depending on the amount of tissue contained. Generating patches, or tiles, in this way is a standard preprocessing step when working with large slide images^17^. Over 17,600, 4096 × 4096 usable patches (i.e., containing at least 10% tissue) can be generated from this dataset, with 25% overlap along x and y axes. For analyses, one option is to utilize a CNN with multiple instance learning (MIL), commonly used for histopathology tissue imaging datasets. Another common technique for this type of image data is to utilize feature-extraction algorithms so that features of importance can be fed into an artificial neural network (ANN) or other type of machine learning model. Users might find it helpful to consider the reference article by Smith, *et al*.^17^, for standard preprocessing and analysis techniques when working with slide imaging.

This dataset provides labels at the patient, slide, and section levels. Researchers who wish to use this dataset can leverage it by adding labelling or annotations from their own institution. Due to the small number of patients, utilizing additional data and transformation-based augmentation are encouraged for studies making predictions at the patient level. For example, classifying patients as achieving remission or not at a specific time point, or as having CD or UC. Additionally, granuloma classification models should account for extreme class imbalance by supplementing with additional data or utilizing data oversampling of the granuloma class using transformations. This dataset may be used, not only for diagnostic predictions, but also for the development of computational tools capable of processing large amounts of imaging data.

## Data Availability

No custom code is necessary for the generation or processing of this dataset. No image pre-processing, normalization, or cropping was applied to the slide section scans. The dataset^11^ can be downloaded from CIL using the PHP script provided at https://github.com/CRBS/CIL_RS/wiki/Download_CHOC_dataset.
